# Screening of the DNA mismatch repair genes *MLH1*, *MSH2 *and *MSH6 *in a Greek cohort of Lynch syndrome suspected families

**DOI:** 10.1186/1471-2407-10-544

**Published:** 2010-10-11

**Authors:** Georgia Thodi, Florentia Fostira, Raphael Sandaltzopoulos, George Nasioulas, Anastasios Grivas, Ioannis Boukovinas, Maria Mylonaki, Christos Panopoulos, Mirjana Brankovic Magic, George Fountzilas, Drakoulis Yannoukakos

**Affiliations:** 1 Molecular Diagnostics Laboratory, I/R-RP, National Center for Scientific Research "Demokritos", Athens, Greece; 2 Laboratory of Gene Expression, Molecular Diagnosis and Modern Therapeutics, Department of Molecular Biology and Genetics, Democritus University of Thrace, Alexandroupolis, Greece; 3 Molecular Biology Research Center HYGEIA "Antonis Papayiannis", DTCA HYGEIA, Athens, Greece; 4 Department of Medical Oncology - A, Metaxa Cancer Hospital, Piraeus, Greece; 5 Theagenion Cancer Hospital of Thessaloniki, Thessaloniki, Greece; 6 Department of Gastroenterology, "Saint Panteleimon" General Hospital, Nikea, Greece; 7 2nd Medical Oncology Department, St. Savas Regional Oncology Hospital, Athens, Greece; 8 Institute of Oncology & Radiology of Serbia, Belgrade, Serbia; 9 Department of Medical Oncology, Aristotle University of Thessaloniki, Papageorgiou Hospital, Thessaloniki, Greece; 10 Hellenic Cooperative Oncology Group, Athens, Greece

## Abstract

**Background:**

Germline mutations in the DNA mismatch repair genes predispose to Lynch syndrome, thus conferring a high relative risk of colorectal and endometrial cancer. The *MLH1, MSH2 *and *MSH6 *mutational spectrum reported so far involves minor alterations scattered throughout their coding regions as well as large genomic rearrangements. Therefore, a combination of complete sequencing and a specialized technique for the detection of genomic rearrangements should be conducted during a proper DNA-testing procedure. Our main goal was to successfully identify Lynch syndrome families and determine the spectrum of *MLH1*, *MSH2 *and *MSH6 *mutations in Greek Lynch families in order to develop an efficient screening protocol for the Greek colorectal cancer patients' cohort.

**Methods:**

Forty-two samples from twenty-four families, out of which twenty two of Greek, one of Cypriot and one of Serbian origin, were screened for the presence of germline mutations in the major mismatch repair genes through direct sequencing and MLPA. Families were selected upon Amsterdam criteria or revised Bethesda guidelines.

**Results:**

Ten deleterious alterations were detected in twelve out of the twenty-four families subjected to genetic testing, thus our detection rate is 50%. Four of the pathogenic point mutations, namely two nonsense, one missense and one splice site change, are novel, whereas the detected genomic deletion encompassing exon 6 of the *MLH1 *gene has been described repeatedly in the LOVD database. The average age of onset for the development of both colorectal and endometrial cancer among mutation positive families is 43.2 years.

**Conclusion:**

The mutational spectrum of the MMR genes investigated as it has been shaped by our analysis is quite heterogeneous without any strong indication for the presence of a founder effect.

## Background

Colorectal cancer (CRC) is the second most common cause of cancer-related deaths in the industrialized countries [1]. Approximately 5 to 10% of all colorectal cancer cases are due to highly penetrant alleles, which are inherited mostly in an autosomal dominant fashion [2].

Lynch syndrome, also referred to as Hereditary Non Polyposis Colorectal Cancer (HNPCC), is the most common hereditary colon cancer syndrome [1]. However, its actual tumour spectrum is quite heterogeneous. Particularly, it confers high susceptibility to the development of cancer in the female reproductive tract [3], while the risk for developing cancer at other organs such as ovaries, stomach, small bowel, brain and urinary tract ranges from 2% to 13% [4]. It is attributed to germline mutations in either of the mismatch repair (MMR) genes: *MLH1*, *MSH2*, *MSH6 *and *PMS2 *[5]. The main function of the MMR gene products is to identify and correct mismatches as well as short insertion or deletion loops, which occur during replication and recombination [6]. In case of disabled MMR machinery, errors resulting to the contraction/expansion of tandemly repeated sequences, known as microsatellites, accumulate. This condition is termed as microsatellite instability (MSI). Detection of MSI has been a useful prescreening laboratory tool for the recognition of suspected Lynch syndrome cases [2,7].

In clinical practice, the diagnosis of Lynch syndrome is mainly based on the Amsterdam criteria (AMS) [8]. However, families of small size or with atypical features, such as later age of onset for CRC, might be missed if only the AMS criteria are taken into consideration. Therefore, the Bethesda guidelines, which include all clinical conditions, have emerged as criteria of suspicion [2,8].

The selection of the actual "high-risk" patients is a matter of significance, as a proper genetic testing should combine sequencing of the MMR gene coding regions with techniques for the detection of large genomic rearrangements. This is so, because no hotspots have been reported in the MMR genes while deletions/duplications account for a substantial part of mutations associated with Lynch syndrome [9-11]. Nevertheless, the presence of founder mutations have been well documented in some populations, thus facilitating the procedure of genetic testing [1].

The aim of this study was to successfully identify Lynch syndrome families and to report the *MLH1*, *MSH2 *and *MSH6 *mutational spectrum within Greek colorectal cancer families. More specifically, the nature and frequency of the pathogenic alterations has been elucidated in order to develop an efficient DNA-based screening protocol for the Greek colorectal cancer patients' cohort.

## Methods

### Patients

The Amsterdam criteria and the revised Bethesda guidelines were used as selection criteria. Ultimately, twenty-four families were subjected for genetic testing, i.e. monitoring of point mutations or large genomic rearrangements in *MLH1*, *MSH2 *and *MSH6 *genes. Seventeen of the selected families fulfilled the Amsterdam criteria while seven families satisfied at least one of the revised Bethesda guidelines. All families were referred to Molecular Diagnostics Laboratory - NCSR "DEMOKRITOS". All patients gave us written informed consent for participation in our study. The study was approved by the Bioethics Committee of the National Centre for Scientific Research "Demokritos" (Reference Number 240/EHΔ/10.8) in agreement with the 1975 Helsinki statement, revised in 1983.

An additional 951 sporadic colorectal cancer cases, collected from Hellenic Community of Oncology (HECOG), were screened for the genomic rearrangement identified, resulting in the deletion of exon 6 of *MLH1 *gene.

### DNA extraction

Total genomic DNA was isolated from peripheral blood lymphocytes following the salt extraction procedure [12]. The quantity and quality of the DNA samples was determined with "NanoDrop" (Thermo Fisher Scientific, MA, USA).

### DNA sequencing

All exons of the *MLH1*, *MSH2 *and *MSH6 *genes, including splice junctions, were amplified by polymerase chain reaction (PCR). The PCR products were purified with "Nucleofast 96 PCR Plates" (Macherey-Nagel, Germany) and then subjected to automated cycle sequencing with the Big Dye Terminator Cycle v3.1 sequencing kit (Applied Biosystems, Foster City, CA) and electrophoresis on an ABI Prism 310 Sequencer (PerkinElmer, Applied Biosystems, Foster City, CA). Primer pairs were designed with "Primer 3" software and are available upon request.

### RNA extraction and RT-PCR

Total RNA was extracted from peripheral blood lymphocytes using Trizol (Invitrogen, Paisley, UK) following standard protocol. 1000 ng of total RNA was reverse transcribed using 60 μM of random hexamers and 10 units of MMLV reverse transcriptase (Roche Diagnostics, Mannheim, Germany) at 50°C for 30 min, followed by enzyme inactivation at 85°C for 5 min. Subsequently, cDNA was amplified on a new PCR reaction using the proper primer set.

### Multiplex Ligation-Dependent Probe Amplification (MLPA)

MLPA was performed with P003 kit (MRC-Holland, Netherlands) according to the manufacturer's instructions. Fragment analysis was conducted on ABI Prism 310 Genetic Analyzer using GeneMapper software (Applied Biosystems, Foster City, CA). 35 peaks corresponding to each exon of the *MLH1 *and *MSH2 *genes, as well as 7 peaks corresponding to DNA sequences outside these genes, were identified. Their migration was calculated according to the GeneScan ROX-500 size standard (Applied Biosystems, Foster City, CA). Decrease of 30 to 50% in the peak area(s) was considered as deletion of the corresponding exon(s), while increase of 30 to 50% as duplication of the corresponding exon(s).

### Long range polymerase chain reaction and breakpoint analysis

150 ng of genomic DNA was amplified in a 50 μl reaction volume using 1,5 mM Mg^+2^, 300 μM of each dNTP, 0.5 U of "Expand Long Range dNTPack" (Roche Diagnostics). Primers were designed between exon 5 and intron 7 of the *MLH1 *gene. Cycling conditions were as follows: 94°C for 2 min, 94°C for 15 sec/62°C for 30 sec {-5°C/cycle}/68°C for 5 min {×14 cycles}, 94°C for 15 sec/55°C for 30 sec/68°C for 5 min {×20 cycles}, 68°C for 15 min. The PCR product was further amplified using various primer pairs in a 20 μl reaction. Cycling conditions were: 95°C for 2 min, 95°C for 25 sec/59°C for 30 sec/72°C for 2 min {×25 cycles}, 72°C for 5 min. The PCR products were finally subjected to sequencing.

### PCR using diagnostic primers

100 ng of genomic DNA was amplified in a 20 μl reaction volume using 1.5 mM Mg^+2^, 100 μM of each dNTP, 1.25 U of Taq polymerase (HyTest Ltd. Intelligate, Finland). Two primer sets were used (F3/R1 & For/Rev ×15 BRAF) in order to achieve the simultaneous amplification of the locus involving the exon 6 deletion and an independent locus used as an internal control [13,14]. Cycling conditions were as follows: 95°C for 3 min, 95°C for 30 sec/59°C for 30 sec/72°C for 45 sec {×35 cycles}, 72°C for 7 min.

## Results

### Mutational analysis

Twenty-four probands were screened for the presence of point mutations and large genomic rearrangements in either *MLH1*, *MSH2 *or *MSH6 *genes. Subsequently, when a pathogenic mutation was identified, family relatives were screened for the presence of the particular mutation. In total, forty-two samples were subjected to genetic testing. MLPA analysis was performed only in probands that tested negative for point alterations. Seventeen families fulfilled the Amsterdam criteria, while the remaining satisfied one or more of the revised Bethesda guidelines (age of onset < 50y, 1^st ^degree relatives diagnosed with CRC).

Twelve out of the twenty-four families found to carry pathogenic mutations in the mismatch repair genes (*MLH1*, *MSH2 *and *MSH6*) studied. Interestingly, all families that carry a pathogenic mutation fulfilled the Amsterdam criteria, whereas none of the families fulfilling at least one of the Bethesda guidelines carry deleterious aberration in the investigated genes.

A detailed family history along with the clinical data of each proband harboring a deleterious mutation is presented on table 1. The pathogenic mutations identified in twelve families are summarized on Table 2.

**Table 1:** Features of families carrying a germline mutation in either of the MMR genes.

Patient ID	Clinical manifestations (age of onset)	Family history	Criteria used for selection
**F33**	Endometrial Ca (40)	mother hysterectomy at 40y-CRC at 70y, 1^st ^brother CRC at 50y, 2^nd ^brother CRC at 32y	AMS II
**F39**	CRC (50)	father stomach Ca at 64y, **sister ovarian Ca at 56y**, one 2^nd ^degree relative (P) brain Ca at 47y, one 2^nd ^degree relative (P) pancreatic Ca at 58y, 2 2^nd ^degree relatives (P) CRC at 52y & 80y respectively	AMS II
**F41**	Endometrial Ca (~45)- CRC (77)	**1 daughter endometrial Ca at 45y- CRC at 58y**,1 grandson died from CRC at 29y, **1 granddaughter < 5 polyps at 33y**, two brothers CRC > 50y, 1 sister endometrial Ca at 26y, 1 niece endometrial Ca at 40y	AMS II
**F74**	CRC (42)- endometrial Ca (64)	mother endometrial Ca- CRC, **brother CRC at 26y**, grandmother (M) CRC, uncle (M) CRC at 37y- CRC at 54y, cousin (M) CRC at 40y	AMS II
**F84**	Endometrial Ca (40)	mother died from pulmonary embolism, grandmother (M) stomach Ca at 60y, 2 2^nd ^degree relatives CRC, 1 2^nd ^degree relative CRC-metachronous endometrial Ca	AMS II
**F263**	CRC (42)- metachronous CRC (47)	**mother endometrial Ca at 50y**, grandfather (M) stomach Ca, 4 2^nd ^degree relatives CRC, 1 2^nd ^degree relative endometrial Ca at 34y	AMS II
**F111**	CRC (42)	father renal Ca-prostate Ca, grandfather (P) CRC, uncle (P) CRC	Revised Bethesda
**F150**	CRC (40)	father CRC at 48y, uncle (P) CRC at 50y	AMS I
**F656**	CRC (59)	father CRC at 41y, 1^st ^sister CRC at 41y, **2^nd ^sister CRC at 65y**, grandmother (P) gynecological Ca at ?y	AMS II
**F1278**	CRC (33)	**father CRC at 56y**, grandmother (P) gynecological Ca at ?y- CRC at ~43y	AMS II
**F68**	CRC (28)- metachronous CRC (44)	father prostate Ca, **brother 4 polyps at 36y**, 1 2^nd ^degree relative CRC at 45y-metachronous CRC at 80y, **1 2^nd ^degree relative duodenum adenoCa at 42y**	Revised Bethesda
**F1376**	Ca cecum (30)	mother CRC at 50y-polyp in the female reproductive tract at ~45y, grandfather (M) CRC at ~80y, 5 2^nd ^degree relatives CRC < 60y, 1 2^nd ^degree relative endometrial Ca at ~50y-metachronous CRC at 65y	AMSII
**1SRB** (Serbian origin)	CRC (49)	father CRC at 60y, 2 brothers CRC at 49 & 59y respectively, 1 sister endometrial Ca at 48y-metachronous CRC at 57y, 1 sister pancreatic Ca at 63y, 1 niece CRC-synchronous stomach Ca at 25y, 2 2^nd ^degree relatives (P) CRC at 50 & 60y respectively, 1 2^nd ^degree relative (P) bladder Ca at 60y	AMSII

* Family members written in bold italic carry the particular mutation running in the family ** **(P): **Paternal, **(M): **Maternal, **CRC: **ColoRectal Cancer, **Ca: **Cancer

**Table 2:** Germline mutations detected in either of the MMR genes.

Family ID	Gene	Location	Nucleotide change	Protein effect	Method used
F1376	*MLH1*	Exon 1	c.116+5G > C	c.116_117ins227nt	Sequencing
F656	*MLH1*	Exon 1	c.116+5G > C	c.116_117ins227nt	Sequencing
F263	*MLH1*	Exon 6	deletion of exon 6	p.Glu153[33]fsX8	MLPA/long range PCR
F33	*MLH1*	Intron 9	c.790+1G > A	skipping of exons 9 and 10	Sequencing
F41	*MLH1*	Intron 9	c.790+1G > A	skipping of exons 9 and 10	Sequencing
					
F74	***MSH2***	**Exon 5**	**c.940C > T**	**p.Gln314X**	**Sequencing**
F84	***MSH2***	**Exon 7**	**c.1237C > T**	**p.Gln413X**	**Sequencing**
F68	***MSH2***	**Exon 7**	**c.1276G > C**	**partial skipping of exon 8**	**Sequencing**
F1278	*MSH2*	Exon 13	c.2089T > C	p.Cys697Arg	Sequencing
F39	*MSH2*	Exon 13	c.2131C > T	p.Arg711X	Sequencing
**1SRB**	***MSH2***	**Exon 13**	**c.2132G > C**	**p.Arg711Pro**	**Sequencing**
					
F150	*MSH6*	Exon 5	c.3202 C > T	p.Arg1068X	Sequencing
					
**F111**	***MSH2***	**Exon 12**	**possible duplication of exon 12**	?	**MLPA**
					

Three of the pathogenic nucleotide substitutions located in exons 5, 7 and 13 of the *MSH2 *gene resulted in a premature stop codon. The nonsense mutations *MSH2*, p.Gln314X (exon 5) and p.Gln413X (exon 7) are novel, to the best of our knowledge. The *MSH2*, p.Arg711X (exon 13) has been reported several times in the LOVD database, as well as in an older study conducted in Greek colorectal cancer patients [15].

**Figure 1:**
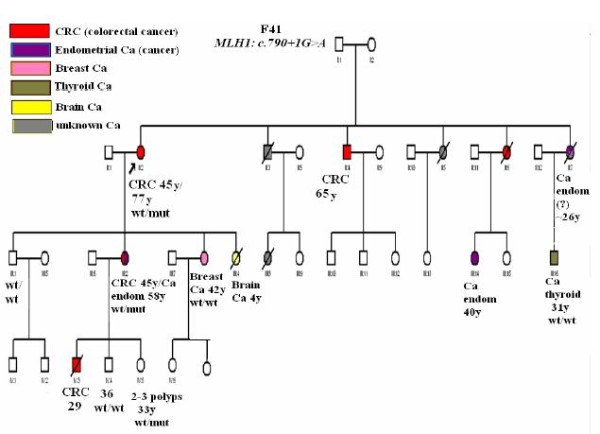
**F41 pedigree**. The F41 family carries the splice donor site alteration (c.790+1G > A) in exon 9 of the *MLH1 *gene. Predominantly colorectal and endometrial cancer cases are encountered across three successive generations, being diagnosed ≤ 50y, thus this family fulfills the Amsterdam criteria II. Furthermore, two metachronous cases of cancer were reported, which is another clinical feature of Lynch syndrome. Particularly, the proband developed 2^nd ^primary colorectal cancer at 77y, while one of her daughters developed colorectal cancer at 45y and endometrial cancer at 58y. Regarding the cases of breast and thyroid cancer reported, it seems that they are not part of Lynch syndrome's clinical manifestations, as the index patients do not carry the particular mutation running in the family. (wt/wt: homozygous for the wild type allele, wt/mut: heterozygous for the mutant allele)

Two splice site changes were detected in four separate families. RT-PCR analysis of the *MLH1*, c.116+5G > C mutation, which is located in intron 1, results in a 227-base pair insertion taking place between exons 1 and 2 (data not shown). The specific mutation has been reported twice in the literature, while both bioinformatic prediction tools and *in vitro *techniques (conversion/cDNA analysis) demonstrated that it activates a cryptic splice site [16,17]. On the other hand, the *MLH1*, c.790+1G > A mutation, which is located in intron 9, induces skipping of exons 9 and 10 [18]. This mutation was identified in two distinct families, one of Greek and one of Cypriot origin. Thirteen different cancer cases have been reported in the Cypriot family, all potentially related to Lynch syndrome. However, one of the proband's daughters, who developed breast cancer at the age of 42 years, does not carry the particular mutation. The detailed family pedigree is shown on figure 1.

Moreover, direct sequencing of DNA from samples F1278 and 1SRB revealed the presence of two discrete missense changes located in exon 13 of the *MSH2 *gene. Exons 7 to 13 of the *MSH2 *gene span the highly conserved ABC-ATPase domain, thus amino acid substitutions in this domain may have a severe impact on the protein's function [19]. The *MSH2*, p.Cys759Arg is characterized as pathogenic in the "MMR Gene Unclassified Variants Database" http://www.mmrmissense.info/. The *MSH2*, p.Arg711Pro mutation, which was identified in a Serbian family, is reported for the first time and its pathogenicity was assessed through bioinformatics tools (Table 3). All the protein web tools used (SIFT, PolyPhen, MAPP-MMR [20]) conclude that it is a deleterious change. In addition, it may influence the splicing process through the abrogation/creation of enhancer/silencer motifs as prediction algorithms for the presence of such sequences have demonstrated (Table 3).

**Table 3:** *In silico *analysis of novel mutation *MSH2*, p.Arg711Pro located in exon 13.

Methods of in silico analysis	Result	Comments
**SIFT score**	0.00 →pathogenic	If SIFT score < .05 then the aa substitution is predicted to affect protein function
**PolyPhen**	Probably damaging (3.071) →pathogenic	If PolyPhen score > 2 then the aa substitution is predicted to affect protein function
**MAPP-MMR**	40.700→pathogenic	If MAPP-MMR score > 4.55 then the aa substitution is predicted to affect protein function
**NNSplice (0.9)**	SD: 1.00/1.00 No SA: 0.95/0.95 Change	Scores predicted for the wt seq/score predicted for the mut seq
**NetGene2 Server**	SD: 0.58/0.62 No SA: 0.71/0.71 Change	Scores predicted for the wt seq/score predicted for the mut seq
Human Splice Finder v2.4 (HSF) ** Rescue ESE **PESE octamers No difference** **ESS (Wang et al)** **Fas-Ess hexamers** **PESS octamers** **IIEs (Zhang et al)** **hnRP motifs**	SD: 74.29/74.4 SA (c.2123):76.67/86.97 SA (c.2126):75.87/79.36 BP:81.68/86.61	Scores predicted for the wt seq/score predicted for the mut seq
	**ESE Finder**:c.2129 SF2/ASF (IgM-BRCA1)→new site **EIEs (Zhang et al): **c.2127→new site **ESE from HSF: **c.2132 9G8)→broken site **Silencer motifs (Sironi et al): **c.2127→new site **Other splicing motifs (Goren et al): **c.2130→new site	

* **SD: **splice donor, **SA: **splice acceptor, **BP: **branch point **aa: **aminoacid

** These algorithms are included in the HSF analysis

The *MSH2*, c.1276G > C involves a novel G to C transversion located at the last nucleotide of the exon 7 of the *MSH2 *gene. According to the *in silico *analysis performed, the wild type splice donor is abolished (Table 4). Furthermore, the majority of the algorithms used for the prediction of enhancer/silencer motifs by the Human Splice Finder (v.2.4) web tool http://www.umd.be/HSF/[21] indicate that the c.1276G > C induces the abrogation or creation of such binding sites.

**Table 4:** *In silico *analysis of novel sequence variations.

Gene (exon/intron)	Nucleotide change	Method	Result	Comments
***MSH2 *(exon 7)**	**c.1276G > A**	**Human Splice Finder (v.2.4)** **(HSF)** (*) **EIEs (Zhang et al) No** **Rescue ESE change** **PESE octamers/no** **ESS (Wang et al) motif** **PESS octamers found** **IIEs (Zhang et al)** **Other splicing motifs** **(Goren et al)**	"wt" donor site broken (84.7/73.68)	Scores predicted for the wt seq/score predicted for the mut seq
			**ESE Finder**: c.1274 (SRp55)→ new site	
			**Rescue ESE: **c.1271/c.1272→ site broken	
			**ESE from HSF: **c.1273/c.1276 (9G8)→site broken	
			**Silencer motifs (Sironi et al): **c.1271/c.1272→site broken	
			**Fas-Ess hexamers: **c.1275→ site broken	
			**hnRNP motifs: **c.1273/c.1274 (hnRNP A1)→site broken	
		**NNSplice (0.9)**	"wt" donor site broken (0.91/-)	Scores predicted for the wt seq/score predicted for the mut seq
		**NetGene2 Server**	"wt" donor site broken (0.83/-)	Scores predicted for the wt seq/score predicted for the mut seq
***MSH2 *(intron 3)**	**c.646/46delC**	**Human Splice Finder (v.2.4)** **(HSF)** (*) **ESE Finder** **Rescue ESE** **PESE octamers** **EIEs (Zhang et al) No** **ESE from HSF change** **Silencer motifs/no** **(Sironi et al) motif** **ESS (Wang et al) found** **IIEs (Zhang et al)** **hnRNP motifs** **Other splicing motifs** **(Goren et al)**	Variation in one of the potential branch points (c.646-48) (79.39/23.83)	Scores predicted for the wt seq/score predicted for the mut seq
			**PESS octamers: **c.646-49 (46.39/87.56)/c.646-46→ new site	
		**NNSplice (0.9)**	SD: 0.90/0.90 No SA: 0.90/0.91 change	Scores predicted for the wt seq/score predicted for the mut seq
		**NetGene2 Server**	SD: 0.76/0.76 No SA: 0.23/0.25 change	Scores predicted for the wt seq/score predicted for the mut seq

(*) these algorithms are included in the HSF analysis

The *MSH6*, c.3202 C > T mutation, located on exon 5 of the gene, has been identified in an individual diagnosed with colorectal cancer at the age of 40. The specific mutation, although has been previously reported, is of interest since it is the first *MSH6 *mutation reported in a Greek family.

### Detection of benign polymorphisms

Eleven intronic variants, three missense and four synonymous changes have been identified in the *MLH1*, *MSH2 *and *MSH6 *genes (Table 5). Two of the reported polymorphisms (*MSH2*, c.646-46delC and *MSH6*, c.3678 A > G) are novel.

**Table 5:** Polymorphisms in either of the major MMR genes.

Gene	Location	Nucleotide change	Protein effect	Families with the variant
*MLH1*	Intron 6	c.545+72T > A	N.A	2
	Exon 8	c.655A > G	p.Ile219Val	12*
	Intron 14	c.1668-19A > G	N.A	6*
*MSH2*	Intron 1	c.211+8G > C	N.A	2
	Intron 1	c.211+9C > G	N.A	4
	**Intron 3**	**c.646-46delC**	**N.A**	**1**
	Exon 6	c.965G > A	p.Gly322Asp	6
	Intron 10	c.1661+12G > A	N.A	14*
	Intron 12	c.2006-6T > C	N.A	9*
*MSH6*	Intron 1	c.261-36 A > G	N.A.	2
	Exon 1	c.116 G > A	p.Gly39Glu	2
	Exon 1	c.186 C > A	p. Arg62Arg	2*
	Exon 2	c.276 A > G	p.Pro92Pro	4*
	Exon 3	c.540 T > C	p.Asp180Asp	4*
	Intron 4	c.3173-101 C > G	N.A.	1
	Exon 5	c.3438+14 A > T	N.A.	8*
	Intron 7	c.3646+29_3646+32delCTAT	N.A.	3*
	Intron 8	c.3802-40 C > G	N.A.	8*
	**Exon 8**	**c.3678 A > G**	**p.Ala1226Ala**	**1**

The *MSH2*, c.646-46delC, located in intron 3 of the gene, has not been previously described. As it was detected in one of our *MLH1*/*MSH2 *mutation-negative patients, we tried to further characterize it through *in silico *analysis (Table 4). The Human Splice Finder (v.2.4) software demonstrated a significant variation in the branch site between the "wild type" and the "mutant" sequence, while both the NNSplice (0.9) http://www.fruitfly.org/seq_tools/splice.html and NetGene2 http://www.cbs.dtu.dk/services/NetGene2/ software packages presented no significant variation in either of the major splice sites. Subsequently, RNA sample was collected from the index patient in order to examine any possible alteration of the above variant on the RNA level. cDNA prepared was amplified with primers spanning exons 2 to 5 of the *MSH2 *gene and revealed the presence of one discernible band corresponding to the wild type transcript (data not shown). Therefore, it is obvious that the *MSH2*, c.646-46delC is a non-pathogenic variation.

### Gene dosage alterations

The eleven families tested negative for point mutations were screened for the presence of large genomic rearrangements using MLPA. The MLPA analysis indicated the presence of genomic rearrangements in two out of twelve families. Particularly, one deletion encompassing exon 6 of the *MLH1 *gene (c.454-?_545+?del) and one duplication involving exon 12 of the *MSH2 *gene (c.1760-?_2005+?dup) have been demonstrated.

**Figure 2:**
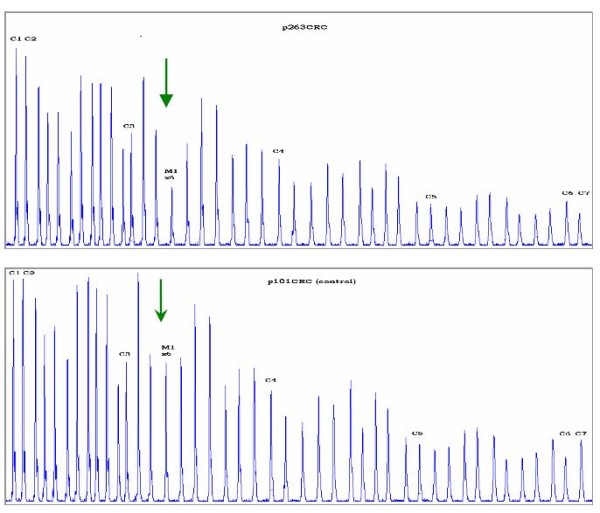
**Identification of the *MLH1*, c.454-?_545+?del by MLPA and its breakpoint analysis**. The electrophoregram of the index proband of 263CRC family in comparison to a control sample is depicted. The two-fold decrease in height of the peak corresponding to exon 6 of the *MLH1 *gene is indicated by the green arrows.

**Figure 3:**
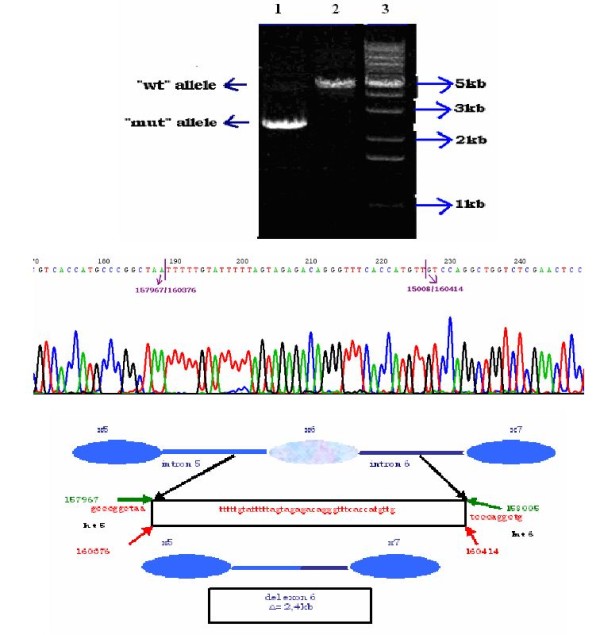
**Breakpoint analysis of the *MLH1*, c.454-?_545+?del**. The products after long range PCR are shown in the upper part of the figure. In the first two lanes corresponding to DNA samples of the 263CRC family members we obtained a discernible band of ~2.5 kb, as well as a fainted band with the expected size of ~5 kb while in the third lane with control DNA sample we got only the band of ~5 kb which corresponds to the expected "wt" allele. Lane 4: 100 bp ladder, lane 5: negative control, lane 6: 1 kB ladder. The PCR product obtained by the combination of F3 and R1 primers was sequenced with R1 and revealed the deletion breakpoints, which are indicated by purple arrows upon the chromatogram. The size and the boundaries of the deletion are depicted schematically in the lower part of the figure.

The *MLH1*, c.454-?_545+?del was confirmed through an individual second MLPA experiment which involved the proband of the family (263), as well as one affected and one unaffected first-degree relative (Figure 2). Subsequently, a long range PCR was performed using a sense primer located in exon 5 and an antisense primer located in intron 7, which preferentially amplified a smaller fragment of about 2.5 kb, instead of the expected wild type fragment of 4937 bp (figure 3). The 2.5 kb fragment was subjected to sequencing in order to uncover the flanking regions of the deleted segment. Then, the 2.5 kb fragment was further amplified using various combinations of intronic primers. Sequencing of the smaller segments produced revealed the boundaries of the deletion, starting between nucleotides 157967-158005 in intron 5 and ends between nucleotides 160376-160414 in intron 6 (GenBank Accession number AC011816). The breakpoints belong to AluSx_C repeats according to the results of RepeatMasker http://www.repeatmasker.org/cgi-bin/WEBRepeatMasker (Figure 3). The exact breakpoints could not be determined due to the presence of two identical 33 bp sequences located within the AluSx_C elements which flank the deletion.

In order to assess the frequency of the *MLH1*, c.454-?_545+?del mutation in the Greek population, 951 unselected sporadic CRC cases were screened using diagnostic primers. The specific deletion was not detected among these samples, indicating that it is not a recurrent mutation in the context of the Greek population. In the case of the duplication of exon 12 of the *MSH2 *gene identified by MLPA, the results have been confirmed by a second individual MLPA experiment, but it was not possible to be confirmed by another method.

## Discussion and Conclusions

Lynch syndrome is a heterogeneous disorder in respects to its molecular basis, as well as its phenotypic expression. Therefore, the selection of the putative mutation carriers as well as the detection of the causative germline alterations is a challenging task. A variety of point mutations, such as substitutions, small insertions/deletions and splice site alterations, as well as large genomic rearrangements have been reported in the international InSiGHT (LOVD) database [10]. Particularly, genomic deletions account for approximately 10% of *MLH1 *and *MSH2 *mutations, while genomic duplications have been observed in approximately 1% of the Lynch syndrome cases [11,22]. Furthermore, the fact that there are multiple susceptible genes that predispose to Lynch syndrome, immunohistochemistry and/or MSI should be used, where possible, as a pre-screening method to successfully identify the high risk families. In our series of experiments, tumor samples from the patients studied were not available and therefore, direct sequencing analysis of the MMR genes was performed.

Nine pathogenic point mutations and one genomic rearrangement have been detected in twelve out of seventeen (70.5%) AMS^+ ^families studied. A 29.5% of AMS^+ ^families that included at least two colorectal or other Lynch syndrome-associated cancer cases remained unresolved. Additionally, no deleterious mutations were detected in patients with young age of onset for CRC, i.e. < 40 years, but with no family history. Our results coincide with observations of previous studies which have demonstrated that a proportion as high as 50% of AMS^+ ^families harbour no pathogenic mutations in either of the major MMR genes, namely *MLH1 *and *MSH2 *genes [9]. Nevertheless, the detection rate among AMS^+ ^families was much higher than in families selected upon Bethesda guidelines, indicating the already reported higher specificity of the modified Amsterdam criteria in clinical practice.

**Figure 4:**
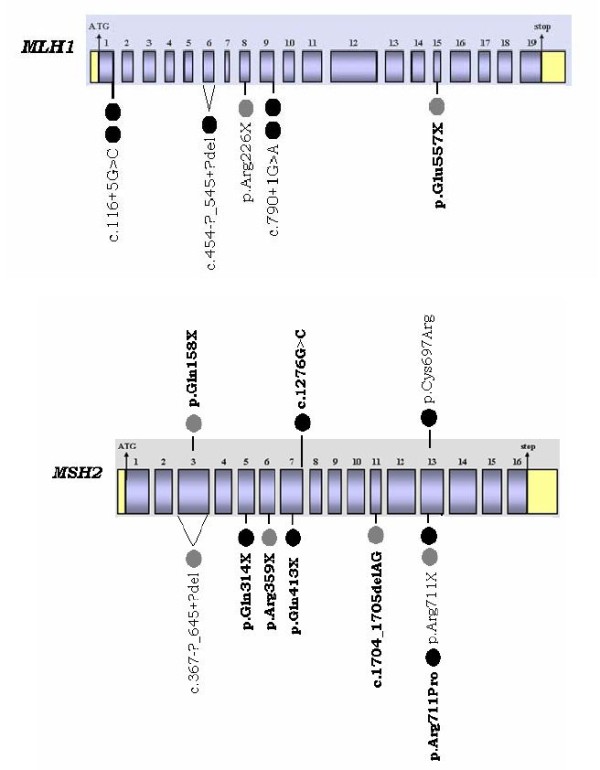
***MLH1 *and *MSH2 *mutational spectrum in two Greek patients' cohorts**. This configuration reviews all the pathogenic germline mutations detected in two Greek patients' cohorts. The black circles represent the families harboring deleterious mutations which were screened in the context of our study, while the grey ones the families carrying pathogenic alterations which were identified by Apessos *et al *[15]. The deleterious aberrations in bold have not been reported in other populations to the best of our knowledge.

Since the particular study includes a relatively small sample size, which can be a limitation when trying to provide a complete mutational spectrum, all available information on Greek putative Lynch syndrome families have been combined [15]. Thirty-three families have been screened for the presence of germline mutations in the *MLH1 *and *MSH2 *genes, while thirteen of them have been screened for the presence of germline mutations in the *MSH6 *gene. Sixteen mutations have been detected in eighteen of these families (Figure 4). To the best of our knowledge, eight of the pathogenic mutations recorded have never been described before.

Genomic rearrangements in the *MLH1 *and *MSH2 *genes represent 6% of the mutations identified in Greece, a percentage that falls within the range reported previously [9,22-25]. More specifically, in a German patients' cohort genomic deletions account for 10.6% of colorectal cancer families, while in the Dutch population genomic aberrations are encountered in 6.5% of AMS^+ ^families [23,24]. If only AMS^+ ^families are taken into account, this proportion rises up to 8.3%, which is in accordance with the observation made by Martínez-Bouzas *et al *[26]. Nonsense mutations and splice site changes seem to prevail among point alterations identified in the Greek patients' cohort. A quite interesting finding of this study is the high rate of novel mutations, which is calculated to be 53.3%, highlighting the distinct heterogeneous nature of the Greek mutational spectrum of the *MLH1 *and *MSH2 *genes.

Despite the aforementioned heterogeneity, two of the mutations described, the *MLH1*, c.790+1G > A transition and the *MLH1*, c.116+5G > C transversion were detected in two distinct families during this study. The *MLH1*, c.790+1G > A has been reported several times in different populations and further analysis is required in order to investigate the possible founder effect of this mutation within the Greek or Cypriot population. The extensive clustering of cancer cases within members of family 41 is of particular interest, since thirteen family members spread across three successive generations were diagnosed with five different cancer types. Interestingly, two of the family members were diagnosed with breast and thyroid cancer, respectively. These two types of cancer are not typical phenotypic features of the Lynch syndrome. Both patients were genotyped and found not to harbour the particular pathogenic mutation, indicating that they were probably sporadic cancer cases. On the contrary, other reports have been able to identify breast cancer in MMR mutation carriers, enhancing the theory that breast cancer can be a rare phenotypic feature of Lynch syndrome [27-31].

On the other hand, the *MLH1*, c.116+5G > C has been reported only once before [16]. There is a possibility that the two families (F656, F1376) harbouring the *MLH1*, c.116+5G > C mutation may be related, but it was not feasible to track down all the family relatives, since most of them live abroad. Further investigations are required in order to elucidate whether the particular alteration has a founder effect within the Greek population.

The aforementioned data underscore the heterogeneity of the *MLH1 *and *MSH2 *mutational spectrum in the Greek population, since each Greek family harbours a distinct deleterious germline mutation, which can be of any type. Furthermore, the first germline pathogenic mutation in *MSH6 *gene in a Greek family is reported here. Therefore, a combination of techniques that will be able to detect both small size alterations within the three genes and large deletions/insertions is required for routine genetic testing of putative Lynch syndrome patients, which is in accordance with other studies.

Moreover, we have achieved to determine the breakpoints of the *MLH1*, c.454-?_545+?del and subsequently to develop diagnostic primers for its rapid detection. This deletion was described initially by Viel and her colleagues [32] and has been reported several times since then. The possible founder effect of the specific mutation within the Greek population has been excluded when 951 unselected colorectal cancer cases were screened and no carriers of the specific mutation have been identified.

It is of great significance to obtain systematically data about the exact nature and frequency of the pathogenic mutations encountered in a particular patients' cohort, in order to customize the genetic testing to the population' s needs, which will eventually result in the reduction of the high cost of genetic testing along with the optimization of the detection rate.

Our results indicate the distinct, heterogeneous nature of Lynch syndrome's associated mutations in a Greek colorectal cancer patients' cohort, as well as the significant contribution of Amsterdam criteria in the identification of putative Lynch syndrome families. Consequently, the compilation of an accurate and detailed family history by the physician is a critical step for the diagnosis of Lynch syndrome.

## Abbreviations

CRC: Colorectal cancer; HNPCC: Hereditary non polyposis colorectal cancer; MMR: Mismatch repair genes; MSI: Microsatellite instability; AMS: Amsterdam; MLPA: Multiplex ligation-dependent probe amplification; PCR: Polymerase chain reaction; InSiGHT: International society for gastrointestinal hereditary tumours;

## Competing interests

The authors declare that they have no competing interests.

## Authors' contributions

GT checked the family histories for their compliance with AMS clinical criteria for the diagnosis of Lynch syndrome, carried out the molecular genetic testing for *MLH1 *and *MSH2*, i.e sequencing and MLPA, as well as the *in silico *and analysis and wrote the paper, FF checked the family histories, carried out a part of the sequencing for *MLH1 *and *MSH2*, carried out all the sequencing for *MSH6 *and revised the paper, RS designed the study and revised the paper, GN referred families to our laboratory after collecting a family history, AAG referred families to our laboratory after collecting a family history, IB referred families to our laboratory after collecting a family history, MM referred families to our laboratory after collecting a family history, CP referred families to our laboratory after collecting a family history, MBM referred a putative Lynch syndrome family of Serbian origin to our laboratory for final diagnosis through genetic testing, GF referred families to our laboratory after collecting a family history as well as blood samples from sporadic colorectal cancer patients collected by HECOG, DY designed the study and revised the paper. All authors have read and approved the final manuscript.

## Pre-publication history

The pre-publication history for this paper can be accessed here:

http://www.biomedcentral.com/1471-2407/10/544/prepub
